# MicroRNA as novel biomarkers and therapeutic targets in diabetic kidney disease: An update

**DOI:** 10.1096/fba.2018-00064

**Published:** 2019-04-10

**Authors:** Qinghua Cao, Xin‐Ming Chen, Chunling Huang, Carol A. Pollock

**Affiliations:** ^1^ Renal Research Laboratory Kolling Institute of Medical Research, The University of Sydney, Royal North Shore hospital St Leonards, Sydney New South Wales Australia

**Keywords:** biomarker, diabetic kidney disease, fibrosis, microRNA, therapy

## Abstract

Diabetic kidney disease (DKD) is a life‐limiting condition characterized by progressive and irreversible loss of renal function. Currently, the estimated glomerular filtration rate (eGFR) and albuminuria are used as key markers to define DKD. However, they may not accurately indicate the degree of renal dysfunction and injury. Current therapeutic approaches for DKD, including attainment of blood pressure goals, optimal control of blood glucose and lipid levels, and the use of agents to block the renin‐angiotensin‐aldosterone system (RAAS) can only slow the progression of DKD. Hence, early diagnosis and innovative strategies are needed to both prevent and treat DKD. In recent years, a novel class of noncoding RNA, microRNAs (miRNAs) are reported to be involved in all biological processes, including cellular proliferation, apoptosis, and differentiation. miRNAs are small noncoding RNAs that regulate gene expression by posttranscriptional and epigenetic mechanisms. They are found to be in virtually all body fluids and used successfully as biomarkers for various diseases. Urinary miRNAs correlate with clinical and histologic parameters in DKD and differential urinary miRNA expression patterns have been reported. Kidney fibrosis is the common end stage of various CKD including DKD. Transforming growth factor‐β(TGF‐β) is regarded as the master regulator of kidney fibrosis, which is likely at least in part through regulating miRNA expression. miRNA are widely involved in the progression of DKD via many molecular mechanisms. In this review, the involvement of miRNA in fibrosis, inflammation, hypertrophy, autophagy, endoplasmic reticulum (ER) stress, oxidative stress, insulin resistance, and podocyte injury will be discussed, as these mechanisms are believed to offer new therapeutic targets that can be exploited to develop important treatments for DKD over the next decade.

## 1 INTRODUCTION

Diabetic kidney disease (DKD), characterized by glomerular hypertrophy, proteinuria, decreased glomerular filtration, and kidney fibrosis, is a major microvascular complication of diabetes.^1^, ^2^ Due to the high prevalence of diabetes, DKD has emerged as an important public health concern as more than a half of patients with type 2 diabetes (T2DM) and one third of those with type 1 diabetes (T1DM) develop DKD.^3^ Consequently, DKD is a major cause of end‐stage kidney disease (ESKD), resulting in death or requiring renal replacement therapy, either kidney transplantation or dialysis.^4^ It thus represents a significant burden on the health system and cost to the community.

Currently, eGFR and albuminuria are used as key markers to define DKD at a specific point in time.^5^, ^6^ eGFR is generally calculated from the serum creatinine measurement with equations that variously also require age, body size, and assigned values based upon sex and race. The two most common equations are Chronic Kidney Disease Epidemiology Collaboration (CKD‐EPI) creatinine equation (2009) and Modification of Diet in Renal Disease Study (MDRD) equation. More recently, calculation of eGFR using other laboratory biomarkers such as cystatin C has emerged with apparent greater accuracy and a different set of CKD‐EPI calculators was established using the result of a cystatin C test. However, equations based on cystatin C overestimate directly measured GFR, while equations based on serum creatinine underestimate GFR.^7^ Also, in elderly persons, the variables affecting creatinine tend to be more pronounced because of comorbid conditions.^8^ Albuminuria strongly predicts progression of DKD, but it lacks specificity and sensitivity for ESKD and progressive decline in eGFR. In T1DM, only about one third of those with microalbuminuria had progressive renal function decline.^9^ In T2DM, a large proportion of those who have renal disease progression are normoalbuminuric.^10^, ^11^, ^12^ To conclude, both eGFR and albuminuria have their limitations as predictors of kidney injury.^13^ Thus, a sensitive and easily detectable biomarker is needed to monitor the decline in kidney function and to separate “progressors” from “non‐progressors” in those with DKD.

Numerous non‐modifiable risk factors for DKD, including ethnicity and inherited genetic difference, have been identified.^14^ Hyperglycemia, advanced glycation end products (AGEs), inflammation via cytokines/chemokines, and aberrant hemodynamics contribute to the above pathological changes.^15^ Hence, current therapies for DKD focus on blood pressure control with inhibitors of the RAAS, on glycemic and lipid control, and lifestyle changes.^16^, ^17^, ^18^ Despite tight control of blood glucose levels with glucose lowing medications, of hyperlipidaemia with statins and of blood pressure with angiotensin‐converting enzyme inhibitors (ACEIs) and angiotensin receptor blockers (ARBs),^19^, ^20^, ^21^ a large proportion of patients develop ESKD.^22^ Therefore, some novel therapeutic strategies are required. Development of novel therapeutic options requires identification of new molecular mechanisms underlying the development of DKD and then targeted development of therapeutics.

Taken together, novel molecular mechanisms underlying DKD should be investigated to design both future biomarkers of disease progression and optimal therapies. With the development of high‐throughput technologies, the important role of epigenetic mechanisms, especially the miRNAs have been explored.^23^, ^24^ Circulating miRNAs are present in body fluids and are known to influence gene expression and regulation.^24^ Abundant expression, lower complexity, tissue specificity, stability, and evolutionary conservation are some of the qualities that make circulating and urinary miRNAs attractive as noninvasive biomarkers to reflect pathophysiological conditions and disease states.^25^ More importantly, a variety of studies have reported the role of miRNAs in the pathology of DKD, thus these small molecules present new possibilities for therapeutic intervention.^23^ Currently, many attempts to downregulate or upregulate miRNAs using one of several delivery approaches in animal models of DKD in vivo have been achieved.^2^ In the future, the control of the expression of miRNA might be developed for patient use.

## 2 miRNA

**Figure 1:**
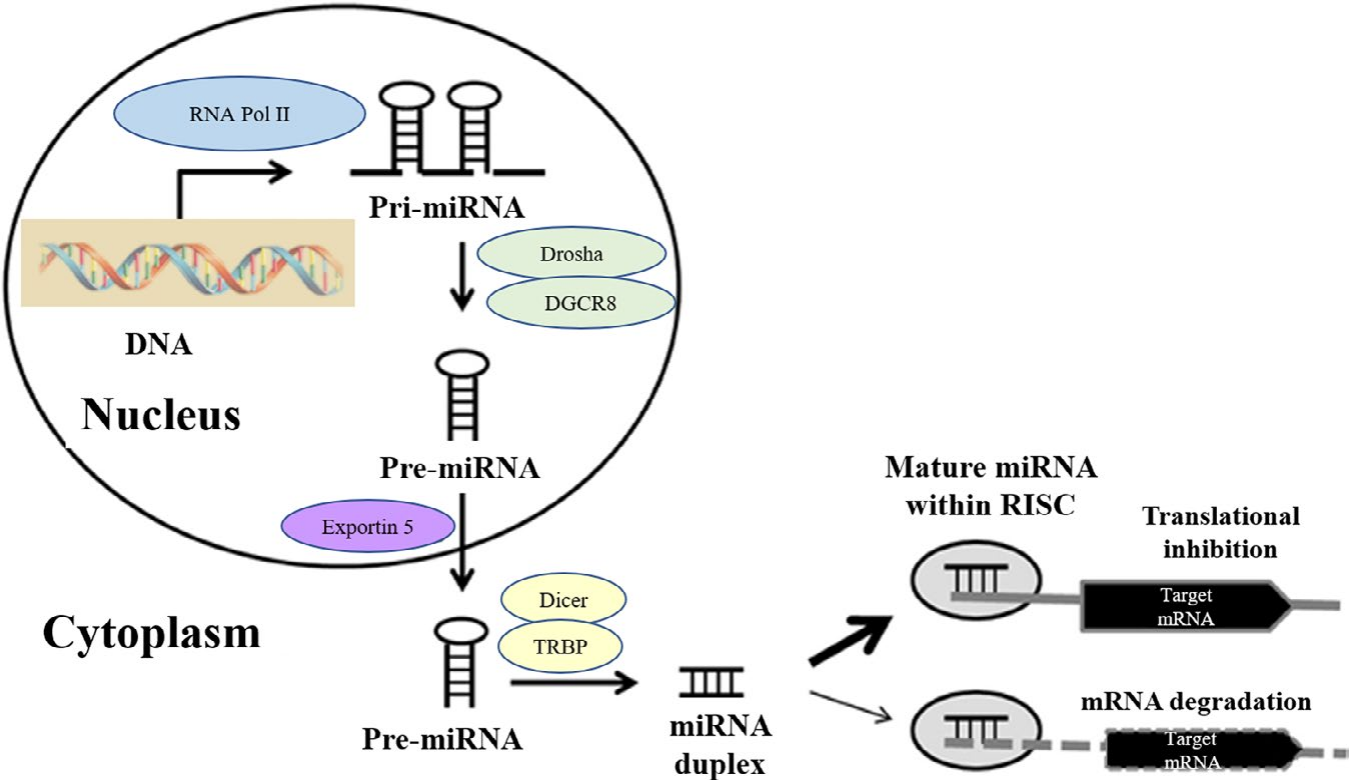
miRNA biogenesis and repression of gene expression

miRNAs (19‐28 nucleotides in length) comprise a novel class of endogenous short noncoding single‐stranded RNA that regulate various cellular processes such as cell death, differentiation, proliferation, metabolism, and pathophysiology of many diseases via the regulation of target gene expression.^23^ Initially, miRNAs are transcribed from DNA into primary‐miRNAs (Pri‐miRNAs), which contain hairpin‐like structures. RNase III Drosha and its binding partner, DiGeorge syndrome critical region gene 8 (DGCR8), bind to the hairpin structures in Pri‐miRNAs and process them into precursor miRNAs (Pre‐miRNAs). Through Exportin 5, Pre‐miRNAs are transferred into the cytoplasm and are processed by another RNase III enzyme, Dicer, in collaboration with transactivating response RNA‐binding protein (TRBP) to generate the mature miRNA duplex. One strand of the duplex goes into RNA‐induced silencing complex (RISC), while the other is degraded. In RISC, mature miRNA recognizes target mRNAs through complete sequence complementarity, resulting in degradation of the target mRNA or more frequently, through incomplete sequence complementarity, resulting in inhibition of translation and protein synthesis (Figure 1).^23^

To date, more than 2500 mature miRNAs have been identified in humans (http://www.miRbase.org), which regulate at least 60% of protein‐coding genes.^26^ Moreover, one specific target gene can be regulated by many different miRNAs, and one single miRNA may alter the expression of a large number of target genes at different levels in a signaling cascade of a particular biological pathway.^27^ Hence, miRNAs can modulate the expression of numerous genes to alter key cellular functions and influence the course of various diseases.

## 3 QUANTIFICATION OF URINARY miRNAs AS BIOMARKERS FOR DKD

### 3.1 Existing biomarkers for DKD

Biomarkers are becoming increasingly important for predicting disease prognosis, enabling personalized therapy (precision medicine), and detecting early therapeutic and adverse responses to drugs. However, the identification, validation, and application of biomarkers are challenging, with several aspects, including understanding the biology of the biomarker and its relevance to disease, and the technological characteristics of the assay used for biomarker measurement.^28^ For DKD, biomarkers are increasingly being investigated for their utility in predicting patients most at risk of decline in kidney function to rationalize and target care.^29^

Clinically, eGFR and albuminuria are used to define the severity of DKD and to loosely identify those at risk of progression to ESKD. However, they may not accurately indicate the kidney function and injury in DKD.^13^, ^30^

Hence, some studies include other risk factors routinely captured in clinical records to predict kidney injury.^31^ These established clinical risk factors involve age, diabetes duration, HbA1c, systolic BP (SBP), albuminuria, prior eGFR and retinopathy status.^31^ However, there have been relatively few attempts to build and validate predictive equations using clinical data that would form the basis for evaluating the marginal improvement in prediction with these biomarkers.^32^, ^33^

Currently, an increasing number of novel biomarkers has emerged to identify both those at risk of DKD and early DKD with the aim of preventing the occurrence of ESKD. These novel biomarkers can be classified as: (a) Glomerular biomarkers: Transferrin, immunoglobulin G, ceruloplasmin, type IV collagen, laminin, glycosaminoglycans(GAGs), lipocalin‐type prostaglandin D synthase (L‐PGDS), fibronectin, podocytes‐podocalyxin, and vascular endothelial growth factor (VEGF); (b) Tubular biomarkers: neutrophil gelatinase‐associated lipocalin (NGAL), α‐1‐microglobulin, kidney injury molecule 1(KIM‐1), N‐acetyl‐β‐D‐glucosaminidase (NAG), cystatin C, and liver‐type fatty acid‐binding protein (L‐FABP); (c) Inflammatory biomarkers: TNF‐α, IL‐1β, IL‐18, interferon gamma induced protein (IP‐10), monocyte chemoattractant protein‐1 (MCP‐1), granulocyte colony‐stimulating factor (G‐CSF), eotaxins, RANTES (regulated on activation, normal T cell expressed and secreted) or CCL‐5; (d) Biomarkers of oxidative: 8‐oxo‐7,8‐dihydro‐2‐deoxyguanosine (8oHdG); (e) Others: podocalyxin, nephrin, AGEs.^13^ Although these biomarkers are potentially useful for the evaluation of DKD, none have been validated to improve clinical decision‐making.^34^ For example, neutrophil gelatinase‐associated lipocalin (NGAL) is a lipocalin iron‐carrying protein of 25 kDa which belongs to the super family of lipocalins. Urinary NGAL concentration has been found to be increased in diabetic subjects compared with healthy controls^35^ and to correlate negatively with eGFR, and positively with CysC, serum creatinine, and urea in patients with T2DM.^36^ Significant increases in urinary NGAL concentration have been demonstrated from normo‐ to micro‐to macroalbuminuric groups of patients with both T1DM and T2DM.^37^ Urinary NGAL correlates positively with glomerular hyperfiltration early in the clinical course of diabetes. However, after adjustment for factors including systolic blood pressure, HbA1c, and diabetes duration, there is no significant correlation between the urinary NGAL concentration and enhanced decline of eGFR in T2D with proteinuria.^37^

A number of “omics” studies have been performed in moderately sized cohorts to identify protein and metabolite biomarkers of DKD. CKD273 is a mass spectrometry‐based method combining data on 273 urinary peptides into a score that has high accuracy in the cross‐sectional classification of eGFR status.^38^ A study of 737 samples obtained at baseline in the Diabetic Retinopathy Candesartan Trials (DIRECT)‐Protect 2 indicated that the CKD273 score was strongly associated with incident microalbuminuria independently of baseline AER, eGFR, and other variables.^39^ Higher CKD273 score at baseline was associated with a larger reduction in ACR in the spironolactone group vs placebo.^40^ However, the interaction between treatment and CKD273 was not statistically significant and the concept that CKD273 will be useful in determining risk of disease progression and may also stratify treatment response is being more definitively tested in the ongoing PRIORITY trial of 3280 participants with T2DM.^41^ Other main “omics” studies include the SUMMIT study using mass spectrometry to measure low‐molecular‐weight metabolites, peptide and proteins (144 in all) as well as 63 proteins by ELISA and Luminex in a prospective design. However, in these global discovery studies, prediction has not been properly assessed on top of available clinical data.^42^

Hence, although these new biomarkers are promising, further studies are needed before establishing in clinical practice and overcoming the problems of specificity and technical variability.^34^ Therefore, it is necessary to explore novel biomarkers to allow accurate identification of kidney injury in DKD and “at risk” individuals.

### 3.2 Advantages of urinary miRNA measurement for kidney function

At present, biopsy is the gold standard diagnostic and prognostic test for kidney disease, but this is a highly invasive and expensive procedure with up to a 3% risk of complications.^43^ Clinically, the majority of patients who develop DKD do not undergo renal biopsy. Given there is an increasing enthusiasm to introduce treatments to reverse metabolic cytopathology before structural pathology develops, early identification of markers of pathology is necessary.

Urine is clearly a biological fluid that can reflect renal pathology.^44^ It can be collected easily, noninvasively, and at low cost. Urinary biomarkers may be elevated in diabetic patients even before the appearance of microalbuminuria and can be used as useful marker for detecting kidney impairment in patients with normoalbuminuria (early DKD).^32^, ^33^, ^34^, ^35^

Circulating miRNAs are relatively stable under different storage conditions such as extreme PH and long‐term room temperature storage, and resistant to RNase activity and repeated freeze‐thaw cycles.^45^, ^46^ Currently available commercial kits provide rapid extraction of total miRNAs from urine, based on the binding of small RNAs to specific material packed in columns.^45^ Up to now, researchers have developed many signal‐amplification strategies for miRNA detection such as hybridization chain reaction, nuclease amplification, rolling circle amplification, catalyzed hairpin assembly amplification, and nanomaterials‐based amplification.^47^ With the development of new technologies, it is now possible to quantify miRNA expression.

These observations, together with the fact that urinary miRNA concentrations have been found to associate with important clinical characteristics, including histopathological diagnosis strongly suggest that urinary miRNAs could be a promising pool of noninvasive biomarkers in DKD.^48^, ^49^, ^50^, ^51^

### 3.3 Urinary miRNAs signature in DKD

miRNAs in urine are released by cells of the nephron and downstream in the urinary tract. They are packed with membrane‐bound extracellular vesicles such as microvesicles, formed by outward budding of the plasma membrane; exosomes, secreted from multivesicular endosomes formed in the endocytic tract, and apoptotic bodies. Urinary miRNAs can also bind to Argonaute proteins and other proteins including high‐density lipoproteins (HDL).^52^ In a study including 80 T2DM patients with normoalbuminuria (n = 30), microalbuminuria (n‐30), or macroalbuminuria (n = 20), Jia et al showed that urinary miRNA‐192 was positively correlated with albuminuria levels and TGF‐β1 expression in patients with normoalbuminuria and microalbuminuria. Receiver operating characteristic (ROC) curve analysis revealed that miRNA‐192 had an area under the curve (AUC) of 0.802, which was better than miRNA‐194 with an AUC of 0.703 and miRNA‐215 with an AUC of 0.757 in discriminating the normoalbuminuric group from the microalbuminuric group, indicating the potential use of urinary miRNA‐192 as a biomarker of the early stage of DKD.^51^

Exosomes in urine originate from most kidney cells and thus are the best‐studied vehicles.^53^ Because exosomes can carry miRNAs to distant target cells, they represent an important mechanism for cell‐to‐cell communication.^54^, ^55^ miRNA‑29 levels in urine exosomes have been proposed as a biomarker of kidney fibrosis 56 but their expression can be dynamic. In a recent study, Mohan et al^57^ reported that urinary exosomal miRNA‐451‐5p level was increased in diabetic rats 3 weeks prior to significant albuminuria and 3 weeks before histological changes of kidney fibrosis. In contrast, renal expression of miRNA‐451‐5p declined and was negatively associated with the indices of renal pathology during the progression of DKD. Thus, urinary exosomal miRNA‐451‐5p may be secreted into the urine by the injured nephron in the early stage of DKD and hold prognostic value as an early and sensitive noninvasive indicator of renal damage.

However, DKD is a multifactorial progressive disease where the pathogenesis is extremely complex involving many different cells, molecules, and factors.^1^, ^2^, ^4^ Also, as many miRNAs have regulatory roles in both DKD and non‐DKD, single miRNA expression might exhibit poor specificity. Hence, there is an increase in studies utilizing urinary miRNA “biomarker panels”—multiple miRNAs measured in combination, to optimize diagnostic sensitivity and specificity as well as being more widely informative of disease processes.^58^, ^59^, ^60^ In a study by Argyropoulos et al,^61^ urine samples were assessed from a historical prospective cohort involving 906 eligible participants recruited from Children's Hospital of Pittsburgh in USA with a diagnosis of T1DM from 1950 to 1980. The urinary miRNA profile was assessed from 30 patients using a Bayesian procedure to normalize and convert raw signals to expression ratios. Urinary miRNA profile of 723 unique miRNAs in the urine of normoalbuminuric T1DM patients who did not develop DKD relative to patients who subsequently developed microalbuminuria were analyzed by qPCR. Eighteen miRNAs were found to be strongly associated with the subsequent development of microalbuminuria, while 15 miRNAs exhibited gender‐related differences in expression. A miRNA signature (miRNA‐105‐3p, miRNA‐1972, miRNA‐28‐3p, miRNA‐30b‐3p, miRNA‐363‐3p, miRNA‐424‐5p, miRNA‐486‐5p, miRNA‐495, miRNA‐5480‐3p and for women miRNA‐192‐5p, miRNA‐720) achieved high internal validity for the future development of microalbuminuria.^61^ However, there are also some limitations in this study. First, the relatively small number of patients makes the precise quantification of changes in expression rather challenging, which is reflected in the large confidence intervals for some of the microRNAs and their apparent lack of prognostic significance. Second, patients in the study have never had renal biopsies so that it is impossible to correlate the urinary miRNA expression with specific tissue pathology.

Recently, there are many studies performing meta‐analysis to screen out potential urinary miRNA biomarker candidates in DKD. For example, Park et al^62^ searched PubMed, Web of Science, and Cochrane Library for the meta‐analysis and suggested that miRNA‐126 and miRNA‐770 family miRNA were significantly dysregulated in both blood and urine from patients with DKD. Hence, they may have important diagnostic and pathogenetic implications for DKD. Another study performed a systematic review of the literature and bioinformatic analyses, indicating six consistently dysregulated miRNAs in DKD patients compared to controls: miRNA‐21‐5p, miRNA‐29a‐3p, miRNA‐126‐3p, miRNA‐192‐5p, miRNA‐214‐3p, and miRNA‐342‐3p. These six miRNAs are involved in pathways related to DKD pathogenesis and may constitute potential biomarkers (Table 1).^63^

**Table 1:** Examples of miRNAs with potential as biomarkers in DKD

miRNAs	Source	Study population	Sample size	Platform	Outcome/DKD stage	Reference
miR‐192	Urinary extracellular vesicles	T2DM patients	80	Real‐time PCR	miR‐192 (AUC = 0.802)/Early stage	51
miR‐29c	Urinary exosome	CKD patients	32	Real‐time PCR	miR‐29c (r = −0.359; *P* < 0.05, AUC = 0.738)/ Tubulointerstitial fibrosis	56
miR‐451‐5p	Urinary exosome	Diabetic rats	43	Pilot small RNA sequencing, Real‐time PCR	Increased miRNA‐451‐5p (>1000‐fold)/ 3‐6 weeks	57
miR‐133b, miR‐342, MiR‐30	Urinary exosome	T2DM patients	156	Bioinformatics analysis, Real‐time PCR	Elevated miR‐133b, miR‐342, and miR‐30a (*P* < 0.001)	58
miR‐2861, miR‐1915‐3p, miR‐4532	Urine	DM patients	145	miRNA profiling, final selection approach, urine miRNA expression analysis, and in situ hydridization	Reduced miR‐2861, miR‐1915‐3p, and miR‐4532 (>10‐fold, *P* < 0.0001)	60
miR‐126, miR‐770	Urine	DM patients	2747	Meta‐analysis	Upregulated miR‐126 (95% CI: 9.96‐862623.21) and miR‐770 (95% CI: 2.37‐44.25)	62

To conclude, urinary miRNAs represent biomarkers with potential not only to augment the utility of albuminuria as a surrogate marker of glomerular filtration barrier (GFB) integrity but may also predict the progression of DKD before the onset of GFB breakdown. However, these studies still have limitations, including the relatively low number of patients recruited and inconsistencies between preclinical and clinical studies. Further investigations that use combined discovery/validation approaches or include larger cohorts are necessary to determine more accurate urinary miRNA expression profiles for early diagnosis and risk stratification in patients with diabetes mellitus.

## 4 THE ROLE OF miRNAs IN THE PATHOGENESIS OF DKD

Strong evidence for the involvement of miRNAs in kidney diseases has come from observations of mice with podocyte specific deletion of Dicer, an essential nuclease involved in miRNA biogenesis, indicating the involvement of miRNAs in kidney diseases.^64^, ^65^, ^66^ In an early study, at least five miRNAs (miRNA‐192, miRNA‐194, miRNA‐204, miRNA‐215, and miRNA‐216a) were considered to have an impact on kidney function as they were identified to be enriched in the kidney compared to other organs.^67^ Thus, the earliest studies to investigate the role of miRNA in kidney dysfunction focused on these miRNAs.

**Figure 2:**
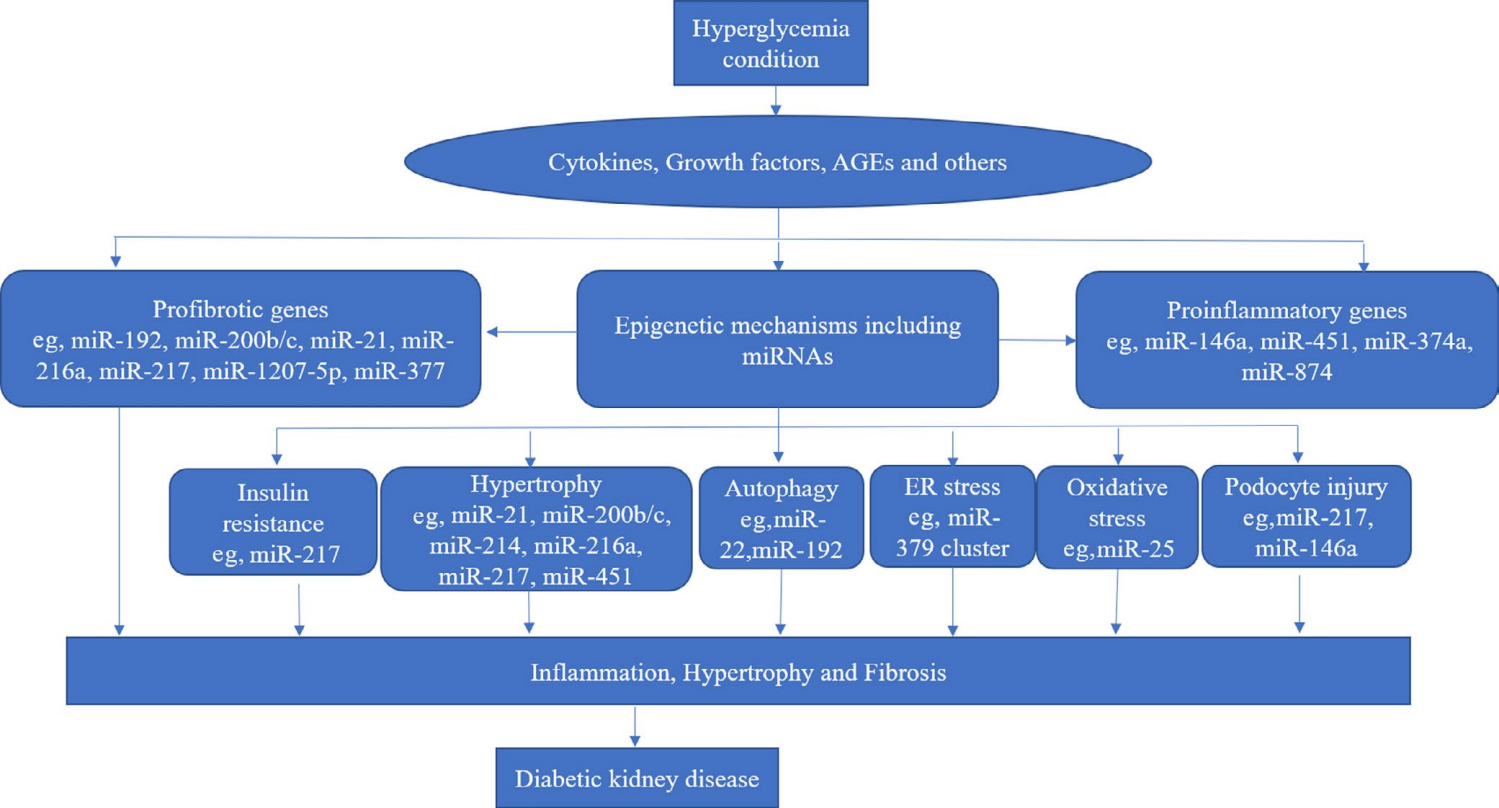
Mechanisms whereby miRNAs influence the pathogenesis of diabetic kidney disease (DKD). Hyperglycemia induces cytokines, growth factors, and dysregulation of miRNAs. miRNAs are involved in the progression of DKD by targeting genes related to fibrosis, inflammation, hypertrophy, autophagy, ER stress, oxidative stress, insulin resistance, and podocyte injury

Over the past 10 years, our understanding of the molecular mechanisms by which diabetic conditions result in damage to the kidney has increased. Diabetic conditions induce inflammation, fibrosis, and hypertrophy in renal cells through various cytokines and growth factors such as TGF‐β1.^5^ The engagement of cytokines and growth factors with their receptors triggers signal transduction cascades that result in the activation of transcription factors to increase expression of inflammatory and fibrotic genes as well as epigenetic states including DNA methylation, chromatin histone modifications, and noncoding RNAs (lncRNAs and miRNAs).^23^ miRNAs induced by diabetic conditions can promote the expression of pathological genes via various posttranscriptional and post‐translational mechanisms.^23^ Kato et al^68^ were the first to describe involvement of a specific miRNA in DKD. They showed that miRNA‐192 is upregulated in vitro in mesangial cells (MCs) and in vivo in glomeruli from type 1 streptozotocin (STZ)‐induced and type 2 db/db mouse models of DKD. Repression of miRNA‐192 may promote collagen deposition in response to TGF‐β.^68^ During the last few years, various miRNAs have emerged as important members in the pathogenesis and progression of DKD through participation in fibrosis, inflammation, hypertrophy, autophagy, ER stress, oxidative stress, insulin resistance, and podocyte injury as summarized in Figure 2.

### 4.1 Effect of miRNAs on kidney fibrosis in the progression of DKD

Kidney fibrosis, characterized by progressive tissue scarring that leads to glomerular and tubulointerstitial fibrosis, is the major pathological feature of ESKD.^69^ Although the precise sequence of molecular events that result in kidney fibrosis has not been completely elucidated, present data indicate that TGF‑β is the master regulator of this process as it acts as the major driver of matrix synthesis, inhibition of matrix degradation, and myofibroblast activation.^69^

TGF‐β isoform, TGF‑β1 acts through a canonical signaling pathway that involves the phosphorylation and activation of Smad2 and Smad3 by the TGF‐β receptor 1(TGFR1, also known as ALK5). Smad4 then binds activated Smad2/3, which enables this complex to translocate to the nucleus and transcribe specific genes. However, TGF‑β1 activation of TGFR1 can also activate a wide variety of Smad‐independent pathways (known as noncanonical signaling) to modify cell function. These non‐Smad pathways include those involving TGF‐β activated kinase 1, PI3K‐AKT, and Rho‐like GTPase signaling pathways.^69^

Recent studies show that TGF‐β1 regulates many miRNAs during the progression of DKD. Compared with nondiabetic control mice, miRNA‐192, miRNA‐200b/c, miRNA‐21, miRNA‐216a, miRNA‐217, and miRNA‐1207‐5p are upregulated in TGF‐β1‐treated murine kidney cells or in glomeruli of mouse models of diabetes.^70^ Functional studies of miRNA‐192 reveal that it can upregulate Collagen 1α (Col 1α) in MCs, which are key genes associated with the pathogenesis of DKD.^68^ miRNA‐192 can also regulate other miRNAs such as miRNA‐216a and miRNA‐217, which are related to cellular hypertrophy in DKD.^71^ miRNA‐200b/c, which are a subset of larger members of the miRNA200 family (miRNA‐200a, miRNA‐200b, miRNA‐200c, and miRNA‐141), are also downstream of miRNA‐192. Moreover, miR200b/c significantly upregulates Col 1α and Col 4α in MCs and diabetic mice.^72^ Wang *el al* showed that miRNA‐377 contributes to inhibition of p21‐activated kinase and superoxide dismutase and enhances fibronectin (FN) accumulation in mouse models of DKD.^73^ miRNA‐1207‐5p, which is upregulated by TGF‐β1, can increase expression of TGF‐β1, PAI‐1, and FN in MCs.^74^

In contrast, miRNA‐200a and miRNA‐141, which are inhibited by TGF‐β1, protect kidneys from fibrosis by suppressing the deposition of ECM and preventing epithelial to mesenchymal transition (EMT), respectively.^70^ Let‐7 family members are also downregulated in cultured human proximal tubular epithelial (HK2) cells treated with TGF‐β1, which induces fibrosis through the TGF‐β1R1 and upregulated collagen expression.^75^, ^76^

Moreover, TGF‐β1 expression can be regulated by miRNAs.^2^ In diabetic mice (STZ and db/db), TGF‐β1 levels are found to be upregulated by miRNA‐192 and miRNA‐200b/c.^72^ In addition, recent studies also demonstrated miRNA‐22 inhibited bone morphogenetic protein‐6 (BMP‐6) and BMP‐7 and further increased TGF‐β1 signaling.^77^ miRNA‐433 increases TGF‐β1 signaling and fibrosis by targeting antizyme inhibitor 1, a regulator of polyamine synthesis.^78^ These miRNA‐regulated circuits may amplify TGF‐β1 signaling to contribute to the progression in DKD. On the other hand, in vitro functional studies have identified type II TGF‐β receptor (TGFRII), Smad3, and TGFβ‐1 itself as miRNA‐23b targets, implying a negative feedback loop regulating TGF‐β‐1 signaling.^79^ miRNA‐29b can also suppress TGF‐β1 expression and thus inhibit TGF‐β1‐induced kidney fibrosis.^80^

### 4.2 Effect of miRNAs on inflammation in the progression of DKD

Augmented inflammation is a hallmark of diabetes,^81^ and this proinflammatory state plays a critical role in the development and progression of DKD.^82^, ^83^ Inflammatory factors such as infiltration of macrophage and inflammatory cytokines and chemokines (MCP‐1, IL‐6, TNF‐α, PAI‐1, CXCR4 etc) can activate myofibroblasts at injury sites in the kidney whilst inducing the differentiation of MCs, glomeruli, and renal tubular epithelial cells into fibroblasts, resulting in enhanced ECM production and deposition, which in turn promote fibrosis.^84^, ^85^, ^86^, ^87^

miRNA‐146a is a known anti‐inflammatory miRNA.^88^ miRNA‐146a expression increased in both peritoneal and intrarenal macrophages in diabetic mice. Mechanistic studies in the miRNA‐146a‐deficient mice showed that miRNA‐146a‐deficiency led to increased expression of M1 activation markers and suppression of M2 markers in macrophages as well as increased expression of proinflammatory cytokines, indicating that miRNA‐146a plays a crucial protective and anti‐inflammatory role during the pathogenesis of DKD.^89^ Activation of nuclear factor‐kappa B (NF‐κB) is associated with inflammation in the progression of DKD.^90^, ^91^, ^92^ miRNA‐451, which is downregulated in the kidneys of diabetic mice and MCs cultured in high glucose conditions, inhibits NF‐κB activity, and downregulated transcription of proinflammatory molecules in MCs.^93^ Yang et al^94^ showed that miRNA‐374a is downregulated in kidney samples from DKD patients. Functional studies indicated that miRNA‐374a suppressed inflammatory response in DKD by inhibition of IL‐6, TNF‐α, and MCP‐1.^94^ Recently, Yao et al^95^ reported that miRNA‐874 was downregulated in STZ‐induced DKD rats. Overexpressing miRNA‐874 with mimics attenuated the inflammatory response by decreasing IL‐6, L‐1β, and TNF‐α.

### 4.3 Effect of miRNAs on hypertrophy in the progression of DKD

Hypertrophy is one key feature of DKD^1^ and the hypertrophy‐related miRNAs have been identified. miRNA‐216a and miRNA‐217 activate Akt (a hypertrophy‐related kinase) by targeting PTEN in the STZ and db/db diabetic mouse model of DKD and MCs treated with TGF‐β1.^71^ In diabetic mice, miRNA‐21 also targets PTEN and activates Akt to contribute to renal hypertrophy.^96^, ^97^ PI3K is the upstream activator of Akt. miRNA‐200b/c activates Akt by targeting the PI3K inhibitor FOG2.^98^ Zhang et al^99^ investigated the role of miRNA‐451 in mesangial hypertrophy and found that miRNA‐451 negatively regulated the expression of Ywhaz (a protein related to activation of p38 MAPK signaling) through binding the Ywhaz 3′UTR. In db/db diabetic mice, miRNA‐451 is downregulated to induce hypertrophy through Ywhaz. miRNA‐34a is increased in MCs under high glucose conditions and db/db mice.^100^ Downregulation of miRNA‐34a can alleviate glomerular hypertrophy through targeting of growth arrest specific 1(GAS1).^100^ Recently, increased expression of miRNA‐214 has been found to be associated with decreased levels of PTEN and enhanced Akt phosphorylation to contribute to renal hypertrophy in renal glomerular MCs and proximal tubular epithelial cells.^101^ miRNA‐181a is upregulated in MCs exposed to high glucose and it downregulates the mammalian target of rapamycin (mTOR) inhibitor Deptor and activates mammalian target of rapamycin complex 2 (mTORC2) to mediate TGF‐β1‐induced glomerular MC hypertrophy.^102^

### 4.4 Effect of miRNAs on autophagy in the progression of DKD

Autophagy, a lysosomal degradation pathway, plays a crucial role in removing protein aggregates and damaged or excess organelles, including mitochondria, to maintain intracellular homeostasis.^103^ Therefore, autophagy may promote cellular health against various stress conditions, including hypoxia, ER stress, or oxidative stress.^104^ Cellular stresses and excess nutrition induced by metabolic dysfunction such as diabetes, impairs autophagy through activation of mammalian target of rapamycin complex 1 (mTORC1) and the reduction of AMP‐activated kinase (AMPK) and Sirt1 activity.^105^, ^106^ Thus, autophagy plays a crucial role in maintaining homeostasis in several organs, especially metabolic organs, and the impairment of autophagy is involved in the pathogenesis of metabolic diseases including DKD.^106^, ^107^

Recently, several miRNAs have been found to influence DKD by regulating autophagy. miRNA‐22 is found to suppress autophagy and inhibition of the endogenous miRNA‐22 increased autophagy and alleviated high glucose‐induced Col 4 and α‐SMA expression in NRK‐52E cells.^108^ Deshpande et al^109^ reported that the expression of autophagy genes was decreased in kidneys of STZ‐induced and db/db diabetic mice. miRNA‐192 inhibitors can reverse the reduction of autophagy genes in these mice. In kidneys of diabetic miRNA‐192‐KO mice, downregulation of autophagy genes was also attenuated. In mouse glomerular MCs with TGF‐β1 stimulation, miRNA‐192 mimic oligonucleotides decreased the expression of autophagy genes, indicating miRNA‐192 as a potential therapeutic target for DKD.^109^

In high glucose‐stimulated podocytes, miRNA‐217 expression was elevated and inhibition of miRNA‐217 can protectively antagonize high glucose‐induced podocyte damage and insulin resistance by restoring the defective autophagy pathway via targeting PTEN.^110^ Several miRNAs have also been implicated in oxidative stress and ER stress in DKD pathogenesis. For example, about 40 miRNAs are included in the miRNA‐379 cluster, which is regulated by ER stress in DKD.^111^ NOX4 is a major catalytic subunit of NADPH oxidase under hyperglycemia. miRNA‐25 inhibitor increased NOX4 mRNA and protein level in STZ‐induced diabetic rats, suggesting that decreased miRNA‐25 expression may upregulate NOX4 to promote oxidative stress and renal dysfunction in rats.^112^

## 5 THE UTILITY OF miRNAs AS NEW THERAPEUTIC TARGETS

### 5.1 Existing therapeutic targets of DKD

The etiology of DKD includes environmental insults, genetic susceptibility, and metabolic and hemodynamic factors resulting in poor glycemic control, hypertension, albuminuria, and excess cardiovascular risk factors.^5^ Thus, the treatment for DKD is mainly aimed at controlling metabolic and hemodynamic abnormalities. The agents for treatment includes the use of traditional anti‐hyperglycemic agents (AHAs) such as metformin or insulin, and RAAS inhibitors including angiotensin‐converting enzyme inhibitor (ACEI), angiotensin receptor blockers (ARBs) or aldosterone antagonists.

Sodium‐glucose linked transporters‐2 (SGLT2) inhibitors represent a new class of AHAs for the treatment of T2DM. The SGLT2 antagonists block the sodium‐coupled energy‐dependent glucose proximal tubular reabsorption, increase glucose excretion, and lower blood glucose levels. They have been shown to decrease glomerular hyperfiltration and albuminuria and thus reduce the progression of diabetic kidney disease.^113^ Although SGLT2 inhibitors are generally well tolerated, there are potential adverse events that healthcare providers and patients should be aware of. Most of these are related to glucosuria and osmotic diuresis.^114^, ^115^ GLP‐1 (glucagon‐like peptide‐1) receptor agonists are also a novel class of AHAs with a glucose‐dependent effect on pancreatic secretion of insulin and glucagon. They mimic the effects of the incretin hormone GLP‐1, which is released from the intestine in response to food intake. There are currently four approved GLP‐1 receptor agonists in the United States: exenatide, liraglutide, albiglutide, and dulaglutide. Evidence from animal studies indicates that GLP‐1 receptor agonists exert protective role in DKD with mechanisms that seem to be independent of their glucose‐lowering effect. Clinical studies support GLP‐1‐mediated renal protection,^116^ but there are some limitations. The most common adverse effect is gastrointestinal in nature, which include diarrhea, nausea, and vomiting.^117^

To conclude, despite numerous attempts to develop more effective drugs for the treatment of DKD, not many treatments have reached clinical use. Therefore, some novel therapeutic strategies are required.

### 5.2 Development of miRNA‐based therapies for DKD treatment

Many drugs targeting the pathogenic signaling such as inflammation and TGF‐β1 for treatment of DKD (mostly through protein‐coding genes) are under development.^118^ However, because of the limited number of protein‐coding genes, noncoding RNAs including miRNAs are attracting more attention as potential new drug targets.^119^ Distinct features of miRNAs including short sequence and their high homology across multiple vertebrate species make them potentially suitable as therapeutic agents.^27^ Many reports have shown that miRNAs are dysregulated in DKD. As listed in Table 2, the therapeutic potential of miRNAs has been well investigated in functional and mechanistic studies using methods to inhibit DKD‐inducing miRNAs or increase kidney‐protective miRNAs.^2^

**Table 2:** Examples of miRNAs as therapeutic targets in DKD

miRNAs	Targets	Study model	Pathological output	Reference
miR‐192	SIP1, Zeb1 miR‐216a, miR‐217 miR‐200b/c	MCs, STZ‐mice, db/db mice MCs, STZ‐mice, db/db mice MCs, STZ‐mice, db/db mice	↑Col1α1 and Col1α2 ↑MC survival, Hypertrophy ↑TGF‐β1, Col1α, Col4α	68 71 72
miR‐216a, miR‐217	PTEN	MCs, STZ‐mice, db/db mice	↑MC survival, Hypertrophy	71
miR‐200b/c	Zeb1	MCs, STZ‐mice, db/db mice	↑TGF‐β1, Col1α, Col4α	72
miR‐377	PAK1, SOD	MCs, STZ‐mice	↑FN	73
miR‐1207‐5p	G6PD, PMEPA1, PDPK1, SMAD7	MCs, RPTEC	↑TGF‐β1, PAI‐1, FN	74
miR‐146a	Traf6, Irak‐1	STZ‐mice	↓TNF‐α, MCP‐1, IL‐1β, IL‐18 ↓Col1a2, Col4a2, PAI‐1, TGF‐β1	89
miR‐451	Ywhaz, p38 MAPK	db/db mice	↓MC proliferation, mesangial hypertrophy	93
miR‐21	PTEN	MCs, db/db mice	↑ Hypertrophy, COL1α2, FN ↑TGF‐β1, NF‐κB	96, 97
miR‐22	PTEN	NRK‐52E cells, STZ‐rats	↓Autophage, Col4, α‐SMA	108

SIP1: Smad‐interacting protein 1; ZEB1: zinc finger E‐box‐binding homeobox 1; PTEN: phosphatase and tensin homolog; PAK1: p21‐activated kinase; SOD: superoxide dismutase; G6PD: glucose‐6‐phosphate dehydrogenase; PMEPA1: prostate transmembrane protein, androgen induced 1; PDPK1: 3‐phosphoinositide‐dependent protein kinase‐1; SMAD7: SMAD family member 7; Traf6: TNF receptor‐associated factor 6; Irak‐1: Interleukin‐1 receptor‐associated kinase 1; Ywhaz: tyrosine 3‐monooxygenase/tryptophan 5‐monooxygenase activation protein zeta; p38 MAPK: p38 mitogen‐activated protein kinases.

Basically, manipulation of the activity of these miRNAs can be achieved by in vivo delivery of mimics to restore miRNA levels or inhibitors to block miRNA function.^120^ miRNA mimics are double‐stranded synthetic oligonucleotides (oligos) that effect the endogenous functions of the miRNA of interest but following chemical modification have increased stability and are efficiently taken up by cells. The most widely adopted strategy so far to block miRNA function is with chemically modified anti‐miRNA oligos (AMOs) designed against the mature miRNA sequence that are stable in circulation and are cell permeable. Kato et al^111^ have reported that a chemically modified oligo inhibits a cluster of nearly 40 miRNAs, in association with a reduction in glomerular ECM and hypertrophy in diabetic mice.

In addition to AMOs, miRNA inhibition can be achieved by expression of miRNA‐target sequences able to capture pathogenic miRNAs (miRNA sponge), short hairpin RNA plasmids to abrogate miRNA expression via RNA interference, miRNA knockout, or using oligonucleotides complementary either to the 3' untranslated region of the target mRNA binding site sequence (masking approach) or to the sequence of the miRNA (erasers).

Locked nucleic acid (LNA)‐modified oligos are one of the most potent modifications for inhibiting miRNA activity specifically.^121^ LNA‐modified anti‐miRNA‐192 specifically and effectively inhibited miRNA‐192, as well as downstream miRNAs (miRNA‐216a, miRNA‐217, and miRNA‐200 family) and p53 and reduced the gene expression of collagen, TGF‐β, and FN in kidneys of diabetic mice.^122^ Transfer of miRNA‐21 knockdown plasmids, which contained LNA‐anti‐miRNA‐21 into the diabetic kidneys of db/db mice at 10 weeks significantly attenuated microalbuminuria, kidney fibrosis, and inflammation at 20 weeks.^123^

Currently, some miRNA‐based therapeutic strategies are being assessed in clinical trials. The first one is a LNA‐anti‐miRNA‐122 (Miravirsen), which targets hepatitis C virus RNA.^124^, ^125^ In a phase 2 study, Miravirsen demonstrated dose‐dependent antiviral activity maintained over 4 weeks (289). Analyses of kidney biopsies showed that the inhibitor of miRNA‑21 (RG‑012) had positive effects in kidney fibrosis based on data from animal models of Alport syndrome although it was paused recently in clinical trial due to the chronic toxicity. Overexpression of miRNA‑29 seems to be a promising anti‐fibrotic approach. miRNA‑29 mimic (MRG‑201) is being assessed in a Phase II trial for the treatment of patients with a predisposition for keloid formation.^126^ Of note, the anti‐fibrotic effect of miRNA‑29 mimic is not specific to skin fibrosis but might be applicable to ESKD with kidney fibrosis including DKD.

The major obstacle to the therapeutic use of miRNAs is an efficient delivery method. Naked miRNAs are quickly degraded by nucleases and cleared via renal excretion.^127^, ^128^ Moreover, miRNA administration may induce innate immune responses, leading to unwanted toxicities.^129^ Because miRNAs are designed to target multiple pathways, they may cause off‐target gene silencing that can induce toxic effects. To avoid any off‐target effects and nonspecific immune response, miRNA should be delivered both efficiently and specifically.

One of the approaches used to overcome the poor stability and immune responses of miRNAs is based on chemical modifications including (a) ribose 2′‐OH group modification, (b) LNA modification, (c) backbone modification, and (d) peptide nucleic acids (PNA) modification, which can reduce the off‐target effects.^130^ Another strategy consists of conjugation with small transport domains such as aptamers and cell‐penetrating peptides. Aptamers are small single‐stranded oligonucleotides with a three‐dimensional structure that can bind to surface receptors with high affinity and specificity to act as drug delivery agents. Aptamers are suitable for targeting as they are noncytotoxic, non‐immunogenic, with superior tissue penetration, easy to modify and cheap.^131^ Conjugation of miRNAs to aptamers has been used to specifically target the nucleic acid to cells expressing the ligands recognized by the aptamer.^132^, ^133^, ^134^ Conjugation to cell‐penetrating peptides enables crossing of cell and endosomal membranes. With the development of nanotechnology, nanocarriers have recently become popular for miRNA delivery to enhance cellular uptake and delivery effectiveness as well as reduce toxicity.^135^ Nanocarriers are safe and require simple manufacturing. Moreover, they are characterized by their low immunogenicity, low cost, and versatility.^136^ In the future, novel therapies might be based on effective and specific delivery of combined miRNA modulators to complement current treatment for DKD.

## 6 CONCLUSIONS

Diabetic kidney disease is one of the most prevalent and life‐threatening complications of diabetes. Early diagnosis of DKD and identification of those likely to progress to ESKD has become highly important as it enables early treatment of patients before overt pathology presenting with proteinuria is evident. Since DKD is a complex, multifactorial disease, a miRNA profile, that includes multiple miRNAs representing distinct biologic pathways may have a better predictive value than a single miRNA. Although miRNA profiling enables researchers to more understand the complex pathways in the progression of DKD, it is still highly controversial if miRNA expression reflects the cause or consequence of the development of DKD. If they represent the consequence, they can serve as biomarkers. If they represent the cause they can serve as treatment targets. A combination of conventional (eGFR and albuminuria) and novel biomarkers (multiple miRNA to reflect the inflammation, fibrosis, hypertrophy, autophagy, ER stress, oxidative stress, insulin resistance, and podocyte injury) is potentially promising in accurately predicting the risk of ESKD. If miRNAs function as the cause of DKD, targeted multi‐miRNA‐based therapies that either restore or block miRNA expression and activity are very attractive.

## CONFLICT OF INTEREST

None.

## AUTHOR CONTRIBUTIONS

Q. Cao: wrote, edited and revised the manuscript; X.‐M. Chen: reviewed, commented and revised the manuscript; C. Huang: reviewed, commented and revised the manuscript; C.A. Pollock: reviewed, commented, revised and finalized the manuscript.
